# Dietary Compositions and Their Seasonal Shifts in Japanese Resident Birds, Estimated from the Analysis of Volunteer Monitoring Data

**DOI:** 10.1371/journal.pone.0119324

**Published:** 2015-02-27

**Authors:** Tetsuro Yoshikawa, Yutaka Osada

**Affiliations:** 1 Department of Forest Vegetation, Forestry and Forest Products Research Institute, Tsukuba, Japan; 2 Laboratory of Biodiversity Science, Graduate School of Agriculture and Life Science, The University of Tokyo, Tokyo, Japan; University of Western Australia, AUSTRALIA

## Abstract

Determining the composition of a bird’s diet and its seasonal shifts are fundamental for understanding the ecology and ecological functions of a species. Various methods have been used to estimate the dietary compositions of birds, which have their own advantages and disadvantages. In this study, we examined the possibility of using long-term volunteer monitoring data as the source of dietary information for 15 resident bird species in Kanagawa Prefecture, Japan. The data were collected from field observations reported by volunteers of regional naturalist groups. Based on these monitoring data, we calculated the monthly dietary composition of each bird species directly, and we also estimated unidentified items within the reported foraging episodes using Bayesian models that contained additional information regarding foraging locations. Next, to examine the validity of the estimated dietary compositions, we compared them with the dietary information for focal birds based on stomach analysis methods, collected from past literatures. The dietary trends estimated from the monitoring data were largely consistent with the general food habits determined from the previous studies of focal birds. Thus, the estimates based on the volunteer monitoring data successfully detected noticeable seasonal shifts in many of the birds from plant materials to animal diets during spring—summer. Comparisons with stomach analysis data supported the qualitative validity of the monitoring-based dietary information and the effectiveness of the Bayesian models for improving the estimates. This comparison suggests that one advantage of using monitoring data is its ability to detect dietary items such as fleshy fruits, flower nectar, and vertebrates. These results emphasize the potential importance of observation data collecting and mining by citizens, especially free descriptive observation data, for use in bird ecology studies.

## Introduction

Birds have diversified their food habits during their evolutionary histories [1]. Determining a bird’s dietary composition and its seasonal shifts are fundamental for understanding the ecology and evolution of a species [2–4], because they are the basic determinants of its niche and are deeply associated with crucial life events such as breeding and migration. Information about the dietary compositions of birds is also essential for understanding the relationships between birds and other organisms, as well as the various ecological functions they play in ecosystems [5–7]. For example, they can act as insect predators, thereby affecting insect populations and their dietary plants [8], facilitate plant reproduction as mutualistic seed dispersers [9] or pollinators [10], and deter plant reproduction as antagonistic predators of seeds and flowers [11,12]. Thus, quantitative evaluations of bird diets are necessary to understand the structure and behavior of food webs or interaction networks.

Despite its fundamental importance, it is generally difficult to determine the dietary compositions of birds. Various methods have been used to estimate dietary compositions, which have their own advantages and disadvantages [4,13]. Stomach analysis is a powerful method for obtaining estimates [14,15], but is usually destructive for individual birds, and it is biased owing to variation in the diet mode and digestibility [13,16]. Analyses of dung or pellets are simple methods, but they are also biased because of variability in the digestibility of food items [17]. Stable isotope analysis may allow us to estimate the trophic positions of species in a food web [18–20], but it is difficult to identify prey species definitively and prior knowledge of the diet of the target species is needed for ecological interpretation. Recent developments in DNA barcoding allow us to determine diet items directly and can detect otherwise undetected items [21,22]; however, it is still difficult to obtain quantitative estimates (but see [23]). Therefore, it is essential to integrate dietary information obtained by several methods for appropriate evaluations of bird diets and their ecological functions [7,17,24].

Direct observation is a simplistic and primitive method for estimating dietary composition of birds [25,26] and does not require specialized instruments and technical analysis, other than a pair of binoculars or a telescope, and is thus viable for non-professional naturalists. This method is less invasive than other methods such as stomach analysis, and thereby applicable for study of threatened species. This method also can provide us the information on actual foraging behavior and location of the birds, which are essential for understanding their complete foraging ecology, and can guide more comprehensive analysis by other methods. However, this method has the disadvantage of being time consuming, and it is biased owing to variation in the detectability and identifiability of food items [16,27].

Here we have developed a method of overcoming the disadvantages of direct observation, by combining the method with a citizen science approach and hierarchical Bayesian modeling. Citizen science is increasingly recognized as an important approach in ornithological research [28,29], and is used mainly for monitoring the broad-scale spatial distributions of birds and long-term changes in their populations [30,31]. The monitoring data produced by citizen scientists have been applied to a limited extent to elucidate some aspects of bird life histories such as breeding [32]. However, these data also have the potential to provide information about various other aspects of bird biology and ecology, including their dietary compositions. Direct observations and records of avian feeding by a large numbers of volunteers could overcome the time-consuming nature of this method and provide sufficient material to evaluate dietary compositions, although we should consider and address the possible biases and limitations of this method.

In this study, we examined the possibility of using long-term volunteer monitoring data as a source of dietary information for 15 resident bird species from 12 families in Japan. The data were a collection from field observations, which were conducted and reported by volunteers of regional naturalist groups in Kanagawa Prefecture, central Japan. Based on these monitoring data, we calculated two types of dietary compositions for each bird species: “raw observation proportions” (ROPs) and “model-aided observation proportions” (MOPs). The ROPs were simply calculated from the identified observations, but the MOPs also considered the bias of unidentified observations using Bayesian models with additional information related to foraging locations. To examine the validity of these two types of dietary compositions estimated from the volunteer monitoring data, we compared the results with the dietary information obtained for focal birds based on stomach analysis methods, which were reported by previous studies. Finally, we characterized the results produced by these two different methods, and we discussed the potentials and limitations of each method for producing better evaluations of avian diets.

## Materials and Methods

### Study site

The study was conducted in lowland areas of Kanagawa Prefecture, central Japan (35°13′ N to 35°67′ N, 138°92′ E to 139°80′ E; 2416 km^2^). The area has a temperate climate with a mean annual rainfall of about 1600 mm and a mean annual temperature of about 16°C. Temperate forest comprises about 40% of the overall prefectural area, followed by urban areas and farmland. More detailed information is provided in a previous study [33].

### Ethics statement

Our analysis of the volunteer monitoring data was permitted by Mr. Shigeya Suzuki, a chief of the Kanagawa Branch of the Wild Bird Society of Japan on June 9, 2012. This volunteer monitoring did not involve the capture and breeding of wild birds; thus, no approval was required for animal care and use.

### Monitoring dataset and the target bird species

We analyzed the data obtained through volunteer monitoring by the members of the Kanagawa Branch of the Wild Bird Society of Japan between 1977 and 2006 [34]. The analyzed data comprised a collection of field observation records, which the members of the branch throughout the prefecture voluntarily documented and sent as paper cards or Microsoft Excel files to the branch office, according to the designated format of the branch. Each observation described the date, location, bird species, number of individuals, bird behavior (e.g., foraging, singing, nesting, and intraspecific and interspecific conflicts), and site environments (e.g., natural forest, urban area, riverbed, and rice field). During the 29-year period, the total number of observers was about 2200, and the total numbers of observation records comprised about 180000 episodes for 380 bird species. In addition, the specific regional observers (currently about 40 members) were obliged to report at least one episode for an observed bird species per month within their own responsible regional blocks (a total of 104 blocks within the prefecture) [33] where they were encouraged to describe the avian behaviors in as much detail as possible. In our previous analysis [35], we confirmed that the observation effort was equivalent for most bird species and the number of records of bird species in the overall dataset was generally proportional to the emergence rate of birds in the prefecture. This emergence rate of a bird is based on 1560 line censuses, which were conducted on a monthly basis by members of the branch at the 13 routes between 1999 and 2008, independent of focal monitoring. The emergence rate of a bird was calculated as the total number of the censuses that recorded the observation of the bird divided by the total number of the censuses, i.e., 1560.

### Classification of dietary items and the foraging locations of birds

Using all of the observation records, we first extracted all records related to foraging by birds, which were obtained from lowland areas (<500 m altitude) of the prefecture, where we excluded highly urbanized areas and seashores. Next, by probing the descriptions in each of those records, we determined a recorded dietary item and foraging location for each record. The dietary items were classified into 11 categories [seed, fleshy fruit, flower, leaf and bud, invertebrate (including insect, spider, and earthworm), fish, amphibian and reptile, bird, mammal, man-made food (e.g., food waste), and unidentified]. We excluded a very few feeding episodes of uncertain plant or animal materials. For each report, the vertical foraging locations were also determined and classified according to the following 10 categories: ground, puddle, tree, tree trunk, tall grass, short grass, bush, in the air (fly catching), man-made structure (e.g., house roof), and unidentified. The foraging location was defined as the place where the bird caught the dietary item directly. Therefore, if a report only described the location where a bird was handling or processing a dietary item, we treated its foraging location as “unidentified.” In some cases, several dietary items and locations were described in one report, which we separated into different episodes. In the further analysis, we excluded observation records from artificial bird feeders and reports that lacked information on dietary items and foraging locations. In this study, we targeted 15 common, terrestrial resident bird species from 12 families, as shown in Table 1. We selected these species because they had large numbers of feeding episodes, which were sufficient to estimate their dietary compositions.

**Table 1:** Basic information for the 15 focal birds and their feeding episodes used in this study.

Common name	Scientific name	Total No. of feeding episodes	Total No. of identified feeding episodes	Proportion of unidentified episodes	No. of monthly feeding episodes											
					Jan.	Feb.	Mar.	Apr.	May	Jun.	Jul.	Aug.	Sep.	Oct.	Nov.	Dec.
Brown-eared bulbul	*Microscelis amaurotis*	1190	1116	0.062	234	173	179	118	43	44	41	42	34	39	88	155
Eurasian tree sparrow	*Passer montanus*	989	461	0.534	115	71	55	66	102	83	69	69	94	83	93	89
White-cheeked starling	*Sturnus cineraceus*	826	267	0.677	107	87	69	60	116	119	59	42	49	27	36	55
Japanese white-eye	*Zosterops japonicus*	782	580	0.258	149	92	109	65	32	26	16	11	24	69	83	106
Japanese pygmy woodpecker	*Streptopelia orientalis*	748	96	0.872	124	89	89	75	56	43	23	14	29	62	58	86
Great tit	*Parus major*	708	279	0.606	107	72	83	47	74	43	24	25	38	53	57	85
Oriental turtle dove	*Streptopelia orientalis*	566	149	0.737	89	69	55	53	53	25	21	11	34	40	49	67
Grey-capped greenfinch	*Carduelis sinica*	504	270	0.464	70	67	45	48	50	41	29	20	13	38	28	55
Carrion crow	*Corvus corone*	497	227	0.543	50	42	37	54	58	41	22	37	34	31	47	44
Varied tit	*Parus varius*	438	272	0.379	59	38	45	40	26	20	10	9	41	70	37	43
Meadow bunting	*Emberiza cioides*	417	155	0.628	86	77	66	26	14	20	7	8	8	16	38	51
Bull-headed shrike	*Lanius bucephalus*	371	241	0.350	86	49	43	22	10	4	7	5	12	26	41	66
Large-billed crow	*Corvus macrorhynchos*	363	257	0.292	35	29	31	25	33	27	23	18	27	37	38	40
Long-tailed tit	*Aegithalos caudatus*	280	68	0.7571	56	41	32	16	22	12	11	2	11	25	20	32
Azure-winged magpie	*Cyanopica cyanus*	129	95	0.2636	16	13	6	7	4	8	7	8	12	9	13	26

English names and nomenclatures follow those reported previously [36].

### ROPs from the monitoring data

For each bird species, we pooled all the feeding episodes across years and divided them by months, and we then calculated the monthly proportion for each of the 11 dietary item categories. We refer to these proportions as the ROPs for the items. Similarly, we calculated the monthly proportions of foraging locations for each bird species.

### MOPs based on Bayesian modeling

**Fig 1:**
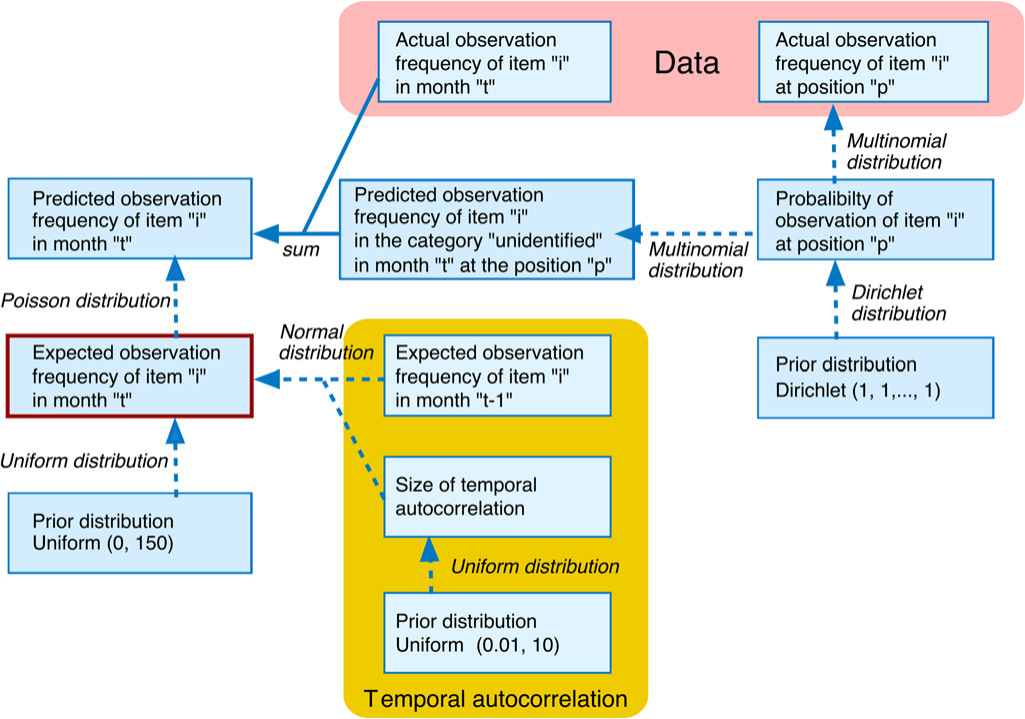
The structure of the Bayesian model developed to infer unidentified feeding episodes by birds.

We used hierarchical Bayesian modeling to consider two potential biases in estimating dietary proportions: 1) variation in the identifiability of dietary items and 2) accidental data fluctuations due to low numbers of observations during some months (Fig. 1). We refer to these estimated proportions as MOPs. Because our modeling details are described in S1 Appendix, we provide a brief explanation on MOPs in this section.

To calculate MOPs, we estimated the expected foraging frequency of each dietary item while considering the existence of “unidentified items” in our data. Our approach to identifying the “unidentified items” was to utilize the relationship between dietary items and foraging locations in the feeding episodes with identified items. We assumed that bird species were observed to forage dietary items at each location randomly with the same probability (by multinomial distributions, i.e., generalization of usual binomial distributions) in both identified and unidentified items. This assumption is marginally too simple but valid because foraging locations are one of the major cause of the variation in the identifiability of dietary items.

In our modeling, we also assumed that expected foraging frequencies were seasonally auto-correlated, i.e., foraging frequency in 1 month was similar to that in neighboring months for each dietary item of each bird species. Introducing the seasonal autocorrelation reduced the bias due to low numbers of observations in some months. We did not assume that the magnitudes of seasonal autocorrelation vary from month to month. Because our analysis used a coarse temporal scale, the change of magnitude of seasonal autocorrelation is likely to introduce relatively little bias to our estimation of the expected foraging frequencies.

All of our Bayesian analyses were conducted using the R software suite (version 3.02 [37]) and Just another Gibbs Sampler 3.3.0 [38]. We used informative prior for all estimated parameters (see S1 Appendix for details). We calculated monthly MOPs of each dietary item after rounding the estimated frequency to an integer.

### Stomach analysis data from previous studies

To examine the validity of the dietary compositions estimated from the monitoring data, we compared these results with the stomach analysis data for birds reported in previous studies of Japanese birds. We collected all the available quantitative dietary data regarding the target birds within the Japanese Archipelago using the following procedures. First, we checked all of the relevant studies in the main ornithological journals of Japan, including Tori (1915–1986), Japanese Journal of Ornithology (1986–2014), Ornithological Science (2002–2014), Strix (1982–2014), Bird Research (2005–2014), Journal of Yamashina Institute of Ornithology (1952–2014), and Bulletin of Applied Ornithology (1980–1990). We also checked agricultural bulletins published between the 1910s and 1950s by the Japanese Ministries of Agriculture, Forestry and Fisheries. In addition, we checked other studies in some cases by tracing citations within the literatures. Next, we selected the studies that included quantitative dietary information related to the focal birds. We focused on stomach analysis data because the selected studies were based almost exclusively on individual-based stomach analysis, apart from a very low number of studies based on direct observations or pellet analysis. In general, these stomach analysis studies used a format that listed a bird species, sample number, sampling location, detected dietary items, and their count numbers (or weights in very few studies), although some studies pooled the results from several individuals in the same month.

For the comparison, we focused on the following eight bird species because abundant monitoring and literature data were available: sparrow, bulbul, two crows, greenfinch, magpie, shrike, and bunting (Table 1). Based on published reports, we calculated the monthly count proportion (CP) for each item. The CP for an item was calculated as the total number of the item detected (pooling across bird individuals from all previous studies) in a month divided by the total number of all items in the same month. The number of parts of plant items (seeds, fruits, flowers, and leaves) and the number of individuals for animal items (invertebrate, bird, fish, amphibian and reptile, and mammal) were counted. When a detected item was uncountable (e.g., resolved or ground after heavy digestion), we counted it as one. Next, for each dietary item, we compared the monthly proportions of the item based on the ROP and the stomach analysis data. In this monthly comparison, we only used the bird—month combinations with more than four individual specimens in the stomach analysis data. To assess the fits between the two types of monitoring-based proportions and the proportions based on the stomach analysis, we calculated root mean square difference between ROPs and CPs, and that between MOPs and CPs. This statistic measures the gap between the two values; a larger value indicates a larger gap.

Note that these stomach analysis-based proportions were also biased in several ways. First, as mentioned earlier, the estimated compositions were inevitably biased due to the variation in diet mode and digestibility. Second, the selected studies only presented the number of each dietary item detected (e.g., the numbers of individual insects or animal species, and the numbers of seed grains or fruits) for each bird. Therefore, the dietary compositions obtained were considerably different from the actual compositions based on the volume, weight, or energy intake. Third, the samples for the stomach analysis were from various localities. Because these sample locations were scattered within the Japanese Archipelago for many of the target bird species, this is expected to average (and offset) the possible bias of locality, and be comparable to the monitoring-based results in Kanagawa Prefecture in central Japan. However, as for the Eurasian tree sparrow, a considerable proportion of the samples were collected from northern parts of Japan, which may cause some bias.

## Results

### General description of the monitoring data

From the overall volunteer monitoring dataset, we obtained 20138 feeding episodes by 120 terrestrial bird species. Among these, we obtained 8903 feeding episodes for the focal 15 resident birds. Feeding episodes were more abundant in winter than in summer, and most abundant in January, followed by February and December, and least abundant in August (Table 1). Among the 15 target birds, the brown-eared bulbul had the most feeding episodes, followed by the Eurasian tree sparrow and white-cheeked starling (Table 1). The proportion of unidentified dietary items during all feeding episodes varied greatly among the 15 birds, ranging from 6.2% for the brown-eared bulbul to 87.2% for the Japanese pygmy woodpecker (Table 1).

### ROPs and foraging location compositions from the monitoring data

**Fig 2:**
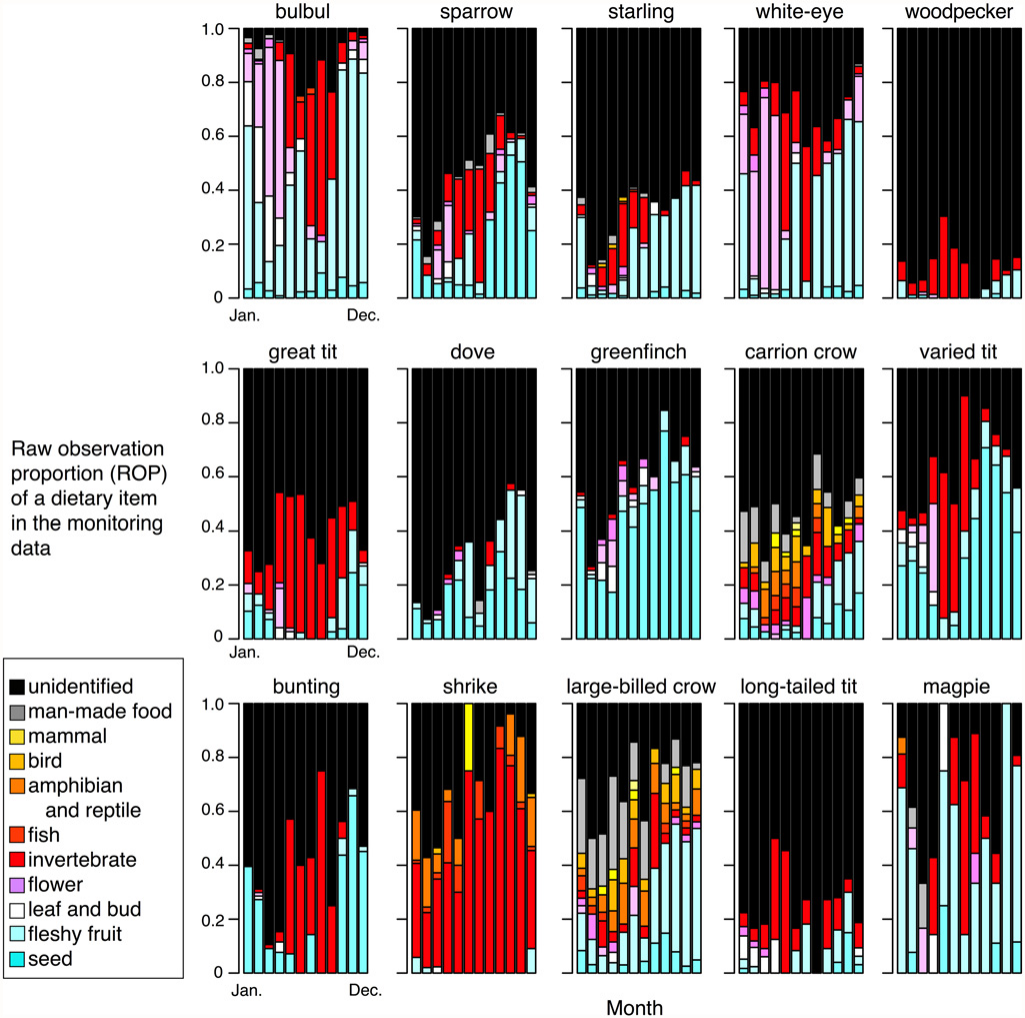
Monthly raw observational proportions (ROPs) of the dietary items for the 15 birds from the volunteer monitoring data. The bar colors correspond to the 11 dietary items. Black corresponds to unidentified episodes.

The ROPs derived from the monitoring data exhibited interspecific and seasonal variations (Fig. 2), although unidentified dietary items comprised large parts of the feeding episodes for some birds (Table 1). In general, the items categorized as “seed”, “fleshy fruit”, or “invertebrate” comprised large parts of the bird diets among their identified feeding items. Noticeable seasonal dietary shifts from vegetable materials (mainly “seed” and “fleshy fruit”) to animal diets (mainly “invertebrate”) were detected in many bird species. We found significant differences within the proportions of the five animal diets within the total bird diets (these being invertebrates, birds, fishes, amphibians plus reptiles, and mammals) between the breeding season (from April to September) and the wintering season (from October to March) for 12 out of the 15 target bird species (*p* < 0.05, one-side Chi-square tests). This trend was particularly obvious in small passerines (e.g., meadow bunting and Japanese white-eye), but the degree was variable between species. By contrast, some birds such as the gray-capped greenfinch and Oriental turtle dove did not exhibit this seasonal shift (one-side Chi-square tests; *p* = 0.50 and *p* = 0.18, respectively), and they utilized vegetable diets constantly. The shrike also utilized animals constantly as its main diet (*p* = 0.09). Vertebrate prey (“amphibian and reptile”, “bird”, and “mammal”) were detected only in two crows, a shrike, a bulbul, and a starling. Human-derived foods comprised part of the diets of two crows and a few birds. In the bulbul and white-eye, flowers comprised large parts of their diet between winter and spring (Fig. 2).

**Fig 3:**
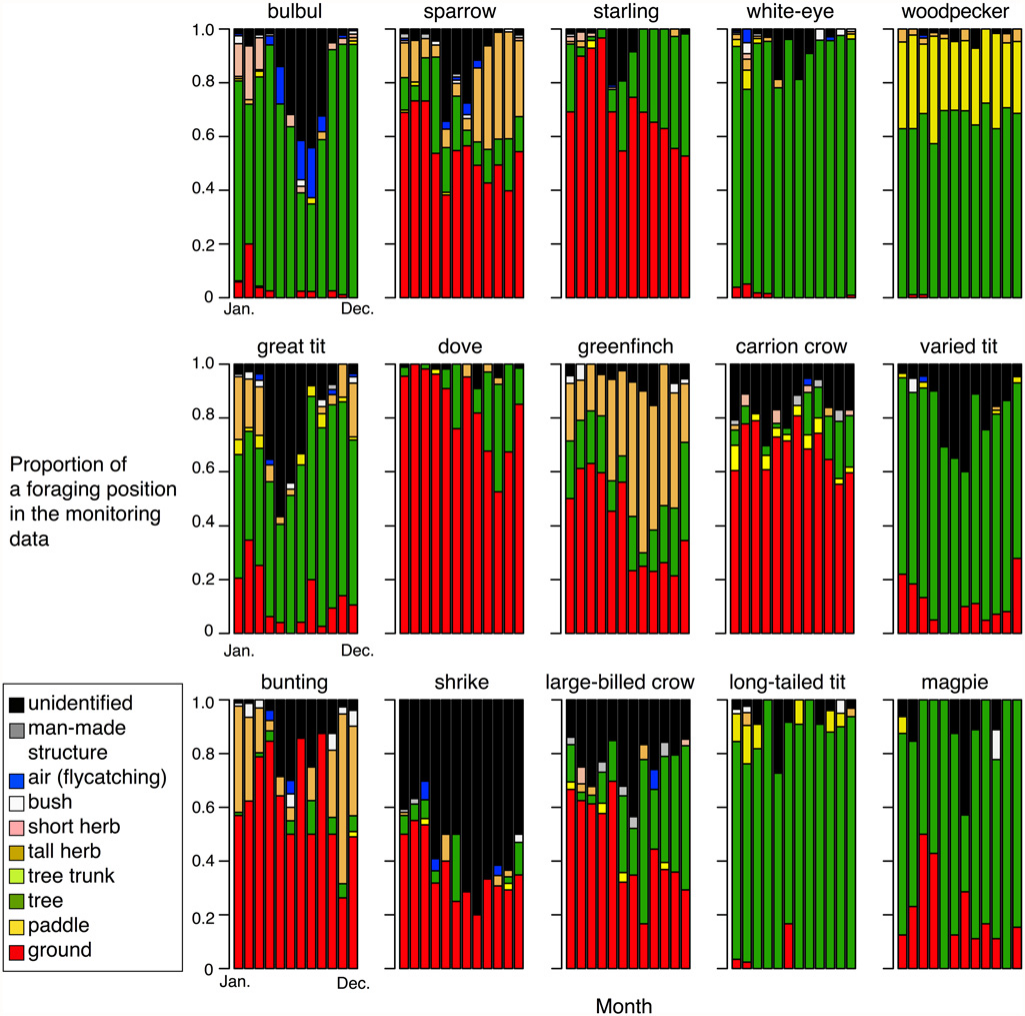
Monthly proportions of foraging locations for the 15 birds from the volunteer monitoring data. The bar colors correspond to the 10 foraging locations.

The compositions of the foraging locations also varied among bird species and seasons (Fig. 3). The results showed that some birds were ground-feeders, such as the sparrow, starling, dove, crows, and shrike, whereas others were tree-feeders, such as the bulbul, white-eye, woodpecker, varied tit, and long-tailed tit. The sparrow, great tit, greenfinch, and bunting utilized high herb plants to some degree. For some birds such as the bulbul, woodpecker, great tit, and varied tit, the proportions of foraging on the ground had a tendency to increase from the breeding seasons to the wintering seasons (one-side Chi-square tests, *p* < 0.01).

### MOPs based on Bayesian modeling

**Fig 4:**
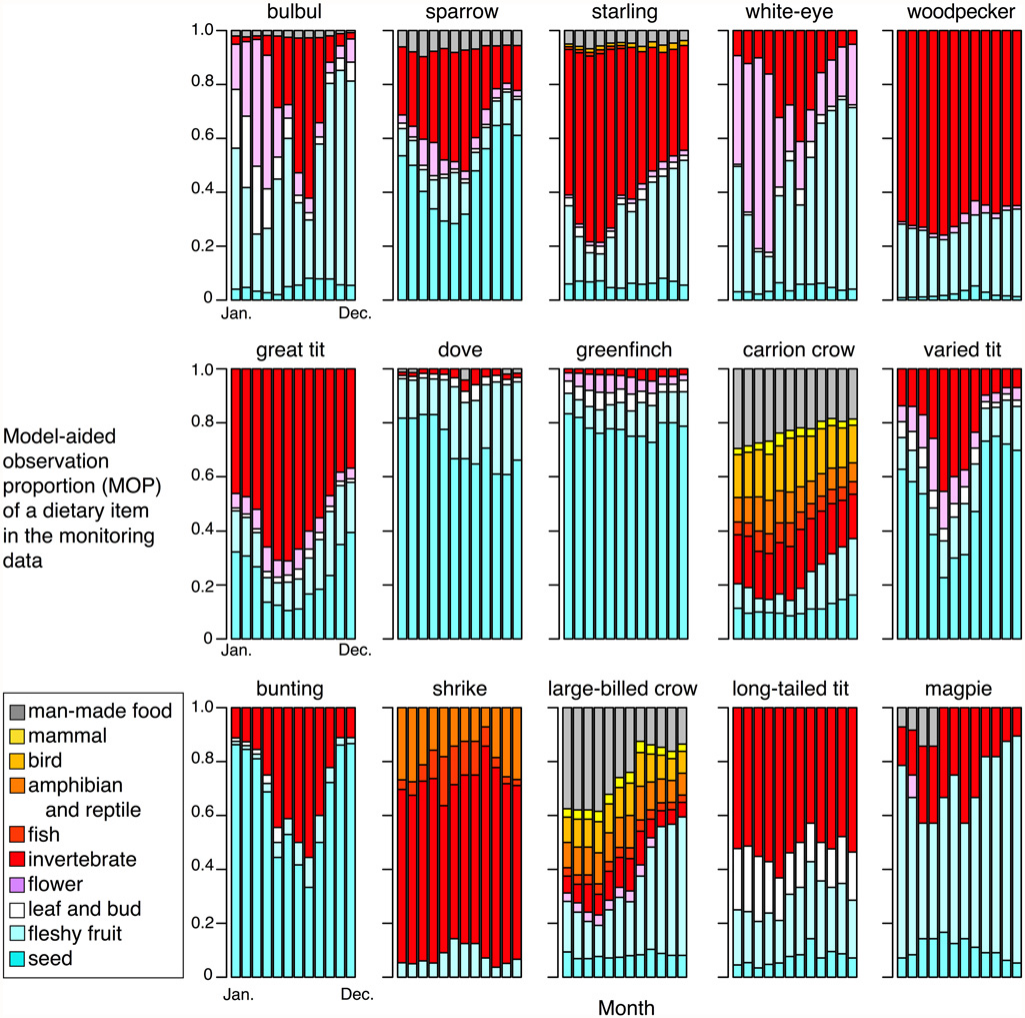
Monthly model-aided observation proportions (MOPs) of the dietary item for 15 birds. The Bayesian modeling approach shown in Fig. 1 was used to infer the types of dietary items for unidentified feeding episodes (black parts in Fig. 2; see Materials and Methods for details).

The MOPs estimated using Bayesian modeling (Fig. 4) were generally similar to the results of the ROPs (Fig. 2), although there were some differences for the birds with large numbers of unidentified items. Overall, the unidentified items tended to be assigned to the items “invertebrate” or “seed.” A major difference in the MOPs compared with the ROPs was the remarkable increase in the vegetative diets of birds, such as the Japanese woodpecker, meadow bunting, and great tit. In these birds, large parts of the unidentified feeding episodes were assigned to “seed” or “fleshy fruit.” By contrast, the unidentified episodes were largely assigned to vegetative diets for the Oriental greenfinch and Oriental turtle dove, which was mainly consistent with their ROPs.

### Comparisons with published stomach analysis data

**Fig 5:**
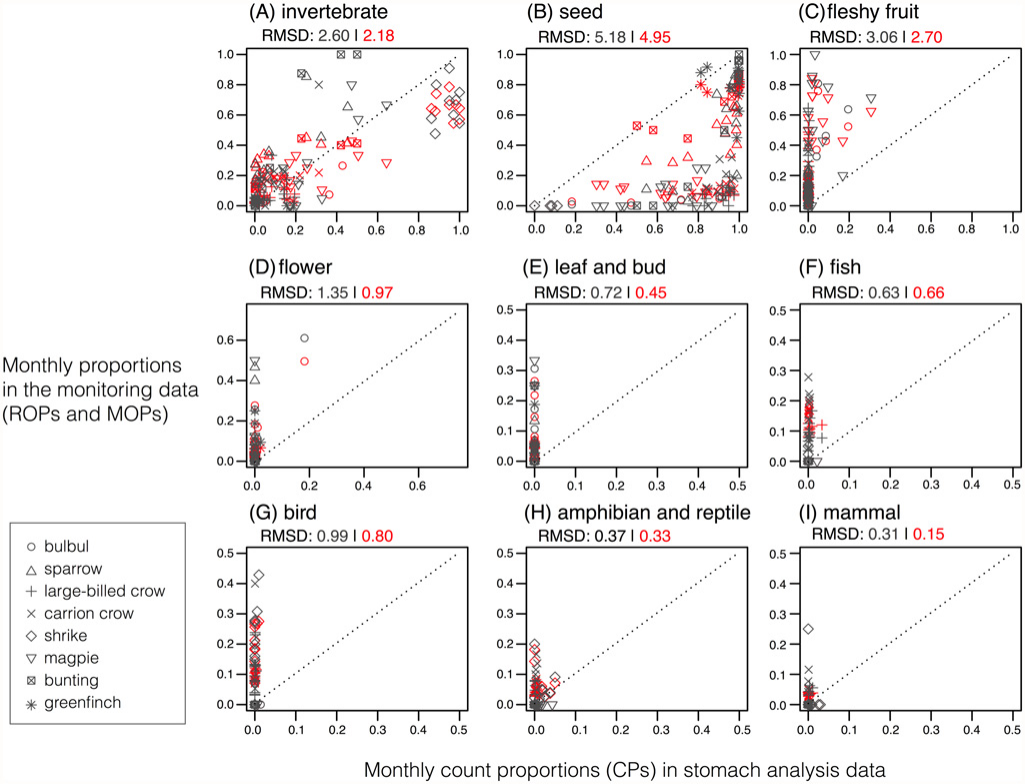
Comparisons of the monthly count proportions (CPs) based on the stomach analyses (*x*-axis) and the monthly observation proportions derived from the monitoring data (*y*-axis) for each of the 9 dietary categories. The Y values with black markers indicate ROPs and those with red markers indicate MOPs. Note that ROPs were recalculated after excluding the proportions of unidentified episodes (i.e., black portions in Fig. 2). The marker shapes correspond to bird species: circle, bulbul; upward triangle, sparrow; cross, large-billed crow; x-mark, carrion crow; diamond, shrike; downward triangle, magpie; square with x-mark, bunting; asterisk, greenfinch. For each diet item, root mean square differences (RMSDs) between ROPs and CPs are presented in black fonts, and those between MOPs and CPs are presented in red fonts.

For each dietary item, we compared the two types of original monitoring data (ROPs and MOPs) with literature-based data (CPs) for eight birds (Fig. 5). The relationships differed among dietary items and the types of monitoring data (Fig. 5). In general, there was a positive relationship between monitoring data and stomach analysis data. This relationship was strengthened more when using MOPs (shown by the red marks in Fig. 5) rather than ROPs. Root mean square differences were smaller between MOPs and CPs than between ROPs and CPs for the 8 of the 9 dietary items, except fish (Fig. 5). For seeds, the ROPs were smaller than the proportions obtained in the stomach analysis data; however, these differences became smaller for the MOPs (Fig. 5B: root mean square difference; ROP = 5.18 vs MOP = 4.95). For the other two plant diets (flowers and leaves), the proportions of the monitoring data greatly exceeded those in the published studies (Fig. 5D, E). This trend was more obvious in the items from the four vertebrate diets (Fig. 5F-I). The vertebrate items comprised a very low proportion in the stomach analysis, whereas the proportions from the monitoring data were much higher.

## Discussion

This study is the first attempt to utilize volunteer monitoring-based observations as resources to determine the dietary compositions of birds and their seasonal shifts. The dietary trends observed in and estimated from the monitoring data (Figs. 2 and 4) were largely consistent with the general food habits determined by previous studies of the focal birds. The estimates based on the volunteer monitoring data successfully detected noticeable seasonal shifts in many of the birds from plant materials to animal diets (mainly invertebrates) during spring—summer. They also appeared to reflect species-specific characteristics in terms of dietary trends. For example, the relative dietary dependency on invertebrates during spring—summer observed in three seed-eating passerines (sparrow, greenfinch, and bunting) based on the monitoring data was consistent with previous stomach analyses, i.e., buntings utilize them more frequently than sparrows [39,40], whereas greenfinches utilize them rarely [41]. The results were also consistent with empirical findings that the white-eye and bulbul depend on flower nectar during spring [42].

The comparison with stomach analysis data (Fig. 5) supports the qualitative validity of the monitoring-based dietary information and the effectiveness of the Bayesian models in improving the estimates. It also demonstrates the advantages and disadvantages of the two different methods. Thus, one advantage of using monitoring data is the ability to detect and evaluate dietary items such as fleshy fruits, flower nectar, and vertebrates. The monitoring-based results demonstrated that the bulbul and white-eye exhibit high dependency on flowers from late winter to spring (Fig. 2), which was not fully evaluated in the stomach analysis (Fig. 5D). Similarly, the monitoring-based data suggests higher proportions of fleshy fruits than the stomach analysis data (Fig. 5C). This is probably because ingested fleshy fruits are often digested; thus, only extracted seeds can be recorded after being separated from the fleshy pulp during stomach analysis (but see [19]). The identification of dietary parts of plants is also difficult by DNA barcoding and stable isotope analysis, thus direct observation methods still have advantages for these items compared with other methods. Another difference between the monitoring data and the stomach analysis data was that the proportion of vertebrate dietary items was much higher in the monitoring data (Fig. 5F-5I). One main cause for this difference was the units used for measurement by the two methods. The stomach analysis data counted the items whereas the monitoring data were based on the number of observed feeding bouts for each item. Thus, the latter may reflect the total time spent consuming an item. The stomach analysis could lead to the dominance of small items, such as seed grains, among the dietary proportions; thus, the dependency on vertebrate diets may have been underestimated in the stomach analysis data used in this study. Another possible cause of the difference is that large-sized items, such as vertebrates, could be easily observed and reported in the monitoring program, thereby overestimated in the ROPs. Given these two possibilities, the actual proportions of vertebrate diets appear to lie between the two estimates.

Our Bayesian modeling approach may also improve the estimation of dietary compositions to some degree. The comparison with stomach analysis data (Fig. 5) showed that MOPs generally had fewer differences from the CPs in the stomach analysis than the ROPs for most of dietary items. For the items where the ROPs exceeded CPs, such as invertebrates, fleshy fruit, flowers, and vertebrates, the modeling reduced the proportions. By contrast, for the “seeds” item where the ROPs tended to be lower than the CPs, the modeling increased the proportion. Although, as mentioned above, stomach analysis data have their own potential biases, the smaller difference between MOPs and CPs can increase confidence in the MOPs. The combination and integration of stomach analysis data and monitoring-based data, as well as other methods, could provide more accurate estimates of bird diets.

Some biases might not have been addressed and controlled in this monitoring-based analysis, e.g., for rare bird foraging behaviors, observers might have tended to record and report rare dietary items rather than common ones, which could have overestimated rare dietary items for a bird. We do not know how much this bias influenced the results, but such bias was shown to be small, at least for rareness of bird species, in our previous analysis [35]. Variation in the levels of expertise among observers is another possible major bias that needs to be considered in citizen science-based approaches [43], but we consider that this bias had little effect on our data because most of the observation records were made by veteran naturalists. Finally, similar to foraging location, the size of a dietary item can also affect detection rate and probability in a way that the item is reported as unidentified in the modeling [16]. Our model could not fully deal with this potential bias. Future refinement of the method is required, including some procedure of calibrating detection rates between dietary items prior to, or during the course of monitoring.

The monitoring data analyzed in this study had unique characteristics because they comprised episode-based observations, which were reported spontaneously by motivated regional naturalists. This additional, detailed information on bird behaviors beyond bird presence/absence records or numbers facilitated our current analysis. Previously, majority of citizen science data used in ornithological studies have comprised bird abundance and/or occurrence data [31], or records of specific events/behaviors such as breeding and migration [32,44]. This is because the data format needs to be as simple as possible to facilitate effective data collection; thus, there is a trade-off between the complexity of the data format and effective data collection by citizens. The inclusion of additional descriptions could reduce the efficiency of data collection and complicate the data structure, but it could be an important source of knowledge related to various aspects of avian natural history, thereby facilitating further ecological research, such as identifying the patterns of interaction networks between birds and other organisms [33,35]. Therefore, data collection and mining of such free description records could be important resources for bird ecology and conservation.

Our method is based on direct observation of bird foraging that is easily conducted by non-professional naturalists. This method is particularly suitable for threatened bird species and those with unknown feeding techniques because direct observation is less invasive than other methods such as stomach analysis (except for nesting and incubating periods), and offers information on actual foraging locations and behaviors of the birds. In addition, this method can effectively distinguish between the dietary preferences of the birds for specific plant parts (i.e., fruits, seeds, leaves, or flowers), which could not be fully determined using DNA barcoding and stable isotope analysis. Thus, this method can be used to evaluate the potential roles that the birds play within plant ecology [5], particularly when detailed descriptions on foraging behavior are recorded. On the other hand, our method can successfully estimate actual dietary compositions of birds under certain conditions. First, the observation of foraging of a target bird species should cover as broad a range of locations that the bird utilizes as possible, so that observed locations are not overly biased to specific locations. Second, considerable focus of observations at each feeding location should be devoted to identifying actual diet. Unidentified dietary items at certain locations can result in excessive extrapolation from the identified items and may result in an invalid estimation. The estimates can be biased, particularly when specific modes of diet items or specific combinations of diet items and locations are expected to have low detection rates [24]. Therefore, consulting and integrating results by other methods is necessary. When collected and treated appropriately, direct observation data by citizens can be an effective method for evaluating foraging ecology of birds, and a fertile source of information for studies on broad aspects of animal ecology.

## Supporting Information

S1 Appendix Details of the Bayesian model used to estimate the model-aided observation proportions (MOPs). (PDF)

S2 Appendix List of the 12 previous studies that included stomach analysis data for the focal birds. The target birds are shown in square brackets.
(PDF)

S1 Dataset Dataset analyzed in this study. (XLSX)

S1 Table Monthly total numbers of specimen from the 12 stomach analysis literatures, listed in S2 Appendix. Square brackets are set for the months with <5 specimen and these months were excluded for comparison in Fig. 5.
(PDF)
