# Supplementary material for: Dietary Compositions and Their Seasonal Shifts in Japanese Resident Birds, Estimated from the Analysis of Volunteer Monitoring Data
Source: PLoS One. 2015 Feb 27;10(2):e0119324. doi: 10.1371/journal.pone.0119324 (PMC4344244; doi:10.1371/journal.pone.0119324)
Supplement: S1 Appendix — (PDF) [file pone.0119324.s001.pdf]

S1 Appendix. Details of the Bayesian model used to estimate the model-aided observation proportions (MOPs).

In our study, we calculated the MOPs using a hierarchical Bayesian modeling. This was because we considered two potential biases in the raw observation proportions (ROPs). The first potential bias was the identifiability of dietary items, i.e., some dietary items were identified more easily than others. In our monitoring data, all of the records were categorized as “unidentified” if volunteer observers could not identify the dietary items accurately. Thus, more easily identified items may have been overestimated without modeling the identifiability. The second bias comprised accidental data fluctuations due to low numbers of observations in some months. To alleviate the influence of data fluctuations on our results, we temporally smoothed the estimated foraging frequency. We explain our Bayesian modeling approach for MOPs in more detail in this supplementary section.

We calculated MOPs from the expected (not observed) foraging frequencies ${R^m}_{i,t}$, which are described as follows:

$$MOP_{i,t} = \frac{{R^m}_{i,t}}{\sum_i {R^m}_{i,t}},$$

where i represents dietary item category and t represents the month. Our data

comprised 10 identified dietary item categories: seed, fleshy fruit, flower, leaf and bud, invertebrate, fish, amphibian and reptile, bird, mammal, and man-made food. If the predicted foraging frequencies ${R^p}_{i,t}$ are defined as observations that have been corrected with respect to the identifiability of dietary items, the predicted foraging frequencies are assumed to follow a Poisson distribution where the expected foraging frequency is the mean,

$$ {R^p}_{i,t} \mid {R^m}_{i,t} \sim \text{Poisson}\left({R^m}_{i,t}\right) . $$

We calculated the predicted foraging frequencies from the observed foraging frequencies ${R^{obs}}_{i,t}$ and unidentified foraging frequencies ${R^u}_{i,p,t}$ (i.e., observed foraging frequencies categorized as "unidentified" at foraging position p),

$$ {R^p}_{i,t} = {R^{obs}}_{i,t} + \sum_p {R^u}_{i,p,t}. $$

We modeled the unidentified foraging frequencies ${R^u}_{i,p,t}$ using information related to the foraging positions to consider differences in the identifiability of dietary items. If $n_{i,p}$ is defined as the observed foraging frequency of dietary item i at position p and $N_p = \sum_i n_{i,p}$, we can estimate the probability $\varphi_{i,p}$ that we observe a bird at position p feeding on dietary item i,

$$n_{i,p} \mid \varphi_{1:11,p}, N_p \sim \text{Multinomial}\left(\varphi_{1:11,p}, N_p\right).$$

In a similar manner, we can describe the unidentified foraging frequencies as

$$R^{u}{}_{i,p,t} \mid \varphi_{1:11,p}, N^{u}{}_{p,t} \sim \text{Multinomial}\left(\varphi_{1:11,p}, N^{u}{}_{p,t}\right),$$

where $N^{u}{}_{p,t}$ represents the unidentified foraging frequency at position p in month t. We assigned an uninformative prior to the probability $\varphi_{i,p}$ according to $\text{Dirichlet}(1, 1, \dots, 1)$.

We temporally smoothed the expected foraging frequencies because these estimates may be affected by data fluctuations due to low numbers of observations in some months. Thus, we assumed that the expected foraging frequencies in neighboring months were correlated according to a normal distribution with amplitude $\sigma_i{}^2$ as the variance,

$$R^{m}{}_{i,t} \mid R^{m}{}_{i,t-1}, \sigma_i{}^2 \sim \text{Normal}\left(R^{m}{}_{i,t-1}, \sigma_i{}^2\right).$$

We assigned an uninformative prior as the expected foraging frequency $R^{m}{}_{i,t}$ and the amplitude of the temporal autocorrelation using $\text{Uniform}(0, 150)$ and $\text{Uniform}(0.01, 10)$, respectively.

The number of iterations for each Markov chain Monte Carlo simulation was

20000, the number of burn-ins was 10000, and the thin interval was 10. The number of chains was three. We confirmed convergence by assessing whether the values of $\hat{R}$ for all estimates were less than 1.1 (Gelman et al. 2003). All of our analyses were conducted using the R software suite (version 3.02; R core team 2013) and Just Another Gibbs Sampler 3.3.0 (Plummer 2003).

Gelman A, Carlin JB, Stern HS, Rubin DB (2003) Bayesian Data Analysis. Chapman and Hall/CRC Press, Boca Raton

Plummer M (2003) JAGS: A program for analysis of Bayesian graphical models using Gibbs sampling. Proceedings of the 3rd International Workshop on Distributed Statistical Computing, Viono hoikuenna
