# Supplementary material for: Dietary Compositions and Their Seasonal Shifts in Japanese Resident Birds, Estimated from the Analysis of Volunteer Monitoring Data
Source: PLoS One. 2015 Feb 27;10(2):e0119324. doi: 10.1371/journal.pone.0119324 (PMC4344244; doi:10.1371/journal.pone.0119324)
Supplement: S2 Appendix — The target birds are shown in square brackets. (PDF) [file pone.0119324.s002.pdf]

S2 Appendix. List of the 12 previous studies that included stomach analysis data for the focal birds. The target birds are shown in square brackets.

Uchida S (1913) Investigation on the relationships between birds and industry in Japan. Special Reports of Agricultural Institute 29: 1–25 [bull-headed shrike]

Momiyama T (1917) Diets of some wild birds. Tori 1: 98–101. [meadow bunting, bull-headed shrike]

Uchida S, Ninobe T, Kuzu S (1922) Investigation on the food habits of sparrows. Ornithological and Mammalogical Report 1: 1–336. Bureau of Agriculture and Commerce. [Eurasian tree sparrow]

Kojima T (1929) Food habits of birds in relation to forest conservation. Bulletin of the Tokyo Imperial University Forest 8: 23–94. [azure-winged magpie, grey-capped greenfinch, meadow bunting, bull-headed shrike]

Uchida S, Kuzu S (1931) Investigation on the food habits of Oriental green finch. Ornithological and Mammalogical Report 5: 1–86, Bureau of Agriculture and Commerce. [grey-capped greenfinch]

Uchida S, Kuzu S (1931) Investigation on the food habits of meadow bunting. Ornithological and Mammalogical Report 5: 87–166. Bureau of Agriculture and Commerce. [meadow bunting]

Yamashina Y (1941) An examination of the food habits of the Japanese birds. Tori 11: 1–46. [grey-capped greenfinch, meadow bunting, and bull-headed shrike]

Kuzu S (1942) Investigation on the food habits of azure-winged magpie. Ornithological and Mammalogical Report 7: 129–242. Bureau of Agriculture and Commerce. [azure-winged magpie]

Inoue M (1950) An examination of the food habits of birds as viewed from the control of may beetles. Tori 13: 9–21. [grey-capped greenfinch]

Ikeda S (1952) Investigations on the relation of wild birds to the industry in Japan. Ornithological and Mammalogical Report 13. Bureau of Agriculture and Forestry. [brown-eared bulbul]

Mizobuchi H (1955) On the food habit of the Brown-eared Bulbul, *Microscelis amaurotis* in the island of Shikoku, Japan. Choju-Schuho 16: 229–240 [brown-eared bulbul]

Ikeda S (1959) On the food habits of some birds belonging to the family Corvidae.

Ornithological and Mammalogical Report 16. Forest Agency. [large-billed crow, carrion crow]
