# Supplementary material for: Dietary Compositions and Their Seasonal Shifts in Japanese Resident Birds, Estimated from the Analysis of Volunteer Monitoring Data
Source: PLoS One. 2015 Feb 27;10(2):e0119324. doi: 10.1371/journal.pone.0119324 (PMC4344244; doi:10.1371/journal.pone.0119324)
Supplement: S1 Table — Square brackets are set for the months with <5 specimen and these months were excluded for comparison in Fig. 5. (PDF) [file pone.0119324.s004.pdf]

S1 Table. Monthly total numbers of specimen from the 12 stomach analysis literatures, listed in S2 Appendix. Square brackets are set for the months with <5 specimen and these months were excluded for comparison in Fig. 5.

	Jan.	Feb.	Mar.	Apr.	May	Jun.	Jul.	Aug.	Sep.	Oct.	Nov.	Dec.	Total No. of used sample
Eurasian tree sparrow	94	67	50	22	239	183	190	212	466	280	487	319	2609
Carrion crow	62	40	37	30	38	65	36	34	49	32	31	30	484
Azure-winged magpie	19	39	39	30	33	29	32	36	47	34	32	31	401
Large-billed crow	22	22	13	15	40	42	31	63	51	36	16	18	369
Brown-eared bulbul	327	70	[0]	8	11	[0]	[0]	[0]	[0]	[0]	[0]	20	436
Grey-capped greenfinch	17	17	39	63	64	[2]	[2]	7	6	10	9	40	272
Meadow bunting	34	27	34	19	11	24	[3]	[2]	15	9	37	6	216
Bull-headed shrike	28	28	6	[2]	7	8	[2]	[1]	7	20	10	5	119
